# Comparison of the accuracy of Japanese synonym identifications using word embeddings in the radiological technology field

**DOI:** 10.1038/s41598-023-49708-8

**Published:** 2023-12-16

**Authors:** Ayako Yagahara, Noriya Yokohama

**Affiliations:** 1 Faculty of Health Sciences, Hokkaido University of Science, 7-Jo 15-4-1 Maeda, Teine, Sapporo, Hokkaido 006-8585 Japan; 2 National Institute of Information and Communications Technology, Osaka, Japan

**Keywords:** Scientific data, Information technology

## Abstract

The terminology in radiological technology is crucial, encompassing a broad range of principles from radiation to medical imaging, and involving various specialists. This study aimed to evaluate the accuracy of automatic synonym detection considering the characteristics of the Japanese language by Word2vec and fastText in the radiological technology field for the terminology elaboration. We collected around 340 thousand abstracts in Japanese. First, preprocessing of the abstract data was performed. Then, training models were created with Word2vec and fastText with different architectures: continuous bag-of-words (CBOW) and skip-gram, and vector sizes. Baseline synonym sets were curated by two experts, utilizing terminology resources specific to radiological technology. A term in the dataset input into the generated models, and the top-10 synonym candidates which had high cosine similarities were obtained. Subsequently, precision, recall, F1-score, and accuracy for each model were calculated. The fastText model with CBOW at 300 dimensions was most precise in synonym detection, excelling in cases with shared n-grams. Conversely, fastText with skip-gram and Word2vec were favored for synonyms without common n-grams. In radiological technology, where n-grams are prevalent, fastText with CBOW proved advantageous, while in informatics, characterized by abbreviations and transliterations, Word2vec with CBOW was more effective.

Clinical standard terms and ontologies are necessary for computing systems to promote unhindered communication, improve workflows, and build applications for quality control, clinical decision support, and clinical trials^1–3^. Natural language allows for a widely varied expressiveness but at the same time may be ambiguous, and jargon and acronyms are used in medical settings^4^. Non-standardized reporting of image observations in patients are a cause of risks in diagnoses with inconsistent communication and inaccuracies in risk estimation by medical experts^5^. Radiological technology is a field covering principles of radiation/magnetic field/ultrasound, engineering for radiation therapy, and imaging technology in addition to medical knowledge. This gives radiological technology a substantial input from engineering among medical fields, and a variety of specialists are involved in this area. Due to the above, the role of terminology in radiological technology is important.

The terminology and ontology are dealing with all the terms in a specific subject field or field of activity by describing the interrelationships between terms. So far, there are a lot of ontologies are created in the medical field, such as the Systematized Nomenclature of Medicine Clinical Terms (SNOMED CT), the International Classification of Diseases (ICD), the Logical Observation Identifiers, Names, and Codes (LOINC), and RadLex. With SNOMED CT there is a comprehensive concept system for healthcare and it is becoming adopted as a standard terminology for electronic health records^3^: ICD is a health statistics coding tool for classification of human diseases, syndromes, and conditions. With LOINC the aim is to provide a means for uniquely identifying the information elements in electronic health records^6^. The first release of LOINC in May 1995 contained only terms for laboratory testing, and LOINC has grown significantly in other fields, including radiology, standardized survey instruments and patient-reported outcome measures, clinical documents, nursing management data, and nursing assessments^7^. RadLex, which is published by the Radiological Society of North America, aims to provide a comprehensive resource for image-related terms, spanning areas such as imaging technologies, image findings, anatomy, and pathology^8^.

Together with these, terminologies in radiological technology has been developed. The International Organization for Standardization (ISO) says that “health terminology is complex and multifaceted, more so than most other language fields,” and “it has been estimated that between 500,000 and 45 million different concepts are needed to adequately describe concepts^9^.” Japanese terminology regarding radiological technology published by the Japanese Society of Radiological Technology has less than 10,000 entries. It is necessary to add terms since there may not be enough terms and to maintain and update the body of recommended terminology continuously.

Developing and maintaining terminologies are difficult work^10^, here particularly as the language of medicine is in a constant flux^11^. Therefore, computational support plays an important role in providing an efficient and accurate terminology expansion. The goal of this study is to edit and maintain the terminology of radiological technology automatically and efficiently. One of the important tasks is to identify synonyms automatically. The same concept may be described in different expressions in the text data. If this happens, a computer will determine that the word is different, and accurate processing will not be possible.

One useful method for automatic synonym detection is the distributed representation such as with Word2vec and fastText, and this is widely applied in the medical field^12^. It expresses words in concepts using hundreds of vectors, and each concept is represented by the activity patterns of multiple vectors, and each vector is also referred to by multiple concepts.

The challenge in the study here is also to apply distributed representation methods to Japanese technical terms, but these methods were originally adopted to the latin alphabet. The Japanese language uses three sets of characters that each have specific grammatical uses—kanji, hiragana, and katakana. Kanji are ideograms which each has their own meanings and correspond to a word. Hiragana and katakana are native sets of syllable characters developed from kanji characters and are used only in Japanese. A Japanese word can be represented in a mixture of kanji, hiragana, and katakana, and it is sometimes shortened. Katakana is also frequently used in transliterations^13^. Moreover, alphabetic characters also found in medical documents as technical phrases and acronyms. For example, “MRI” can be represented in Kanji as "磁気共鳴画像(kakujikikyoumeigazou)" or as a combination of Kanji and abbreviation like “MR画像(MR-gazou).” The term "X-ray" can be translated in Japanese as “X線(x-sen),” which only incorporates the word "ray," as "エックス線(x-sen)" using a transliteration of katakana and kanji, or as "レントゲン線(Rentogen-sen)," named after its discoverer. Given this, synonymous terms in Japanese can differ based on script selection, loanword integration, and context. As a result, expressions in Japanese are intrinsically more intricate than those in Latinate languages. While research on synonym identification using distributed representations in Japanese exists^14–18^, the nuances of complex expression patterns remain underexplored. It is, therefore, crucial to discern the advantages and limitations of distributed representations for managing terminology in radiological technology.

The purpose of this study was to evaluate the accuracy of automatic synonym identification in expression patterns using distributed representations in the field of radiological technology.

## Methods

**Figure 1:**
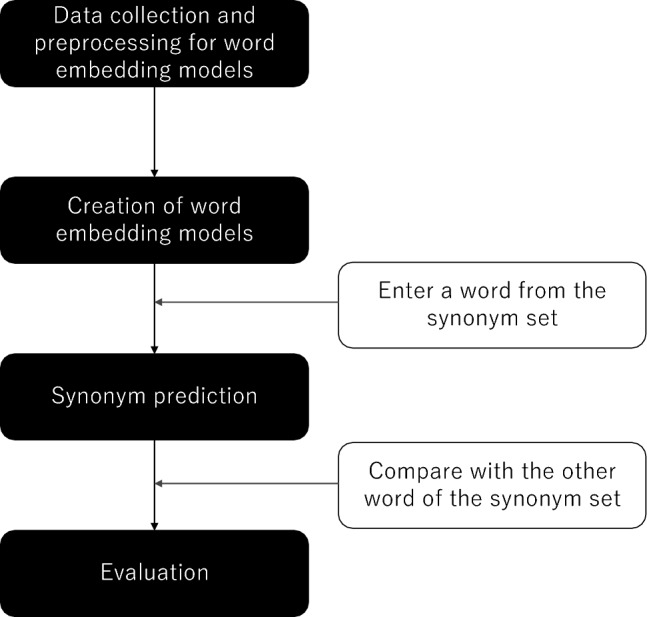
Study flow.

The methodology of this study encompassed several stages: data collection and preprocessing, the development of word embedding models, the formation of synonym sets, prediction of synonyms, and finally, evaluation, as illustrated in Fig. 1.

### Data collection and preprocessing

We collected data from 337,479 abstracts (206 MB), published from 1980 to the data collection date (June 20, 2019), from the Ichushi-Web, a Japanese database^19^. Ichushi-Web archives approximately 400,000 periodicals published in Japan annually. This comprehensive collection includes journals from academic societies, medical publishers, university bulletins, and spans across disciplines such as medicine, dentistry, pharmacy, nursing, and their related fields. To date, the database boasts more than 15 million periodicals. When collecting data, we used “diagnosis imaging” as a keyword and descriptors which are synonyms of the keyword (Table1).

**Table 1:** List of descriptions.

Descriptions
X線像強調 (Radiographic Image Enhancement)
インターベンショナルラジオロジー (Radiology, Interventional)
画像診断 (Diagnostic imaging)
コンピュータ支援画像診断 (Diagnosis, Computer-Assisted)
MRI (Magnetic Resonance Imaging)
出生前診断 (Prenatal Diagnosis)
泌尿器系診断 (Diagnostic Techniques, Urological)
放射性核種イメージング (Radionuclide Imaging)
シネMRI (Magnetic Resonance Imaging, Cine)
コンピュータ支援放射線画像解析 (Radiographic Image Interpretation, Computer Assisted)
テレラジオロジー (Teleradiology)
乳房超音波診断 (Ultrasonography, Mammary)
全身イメージング (Whole Body Imaging)
呼吸同期イメージング (Respiratory-Gated Imaging Techniques)
心臓同期イメージング (Cardiac-Gated Imaging Techniques)
心臓画像診断 (Cardiac Imaging Techniques)
マルチモーダルイメージング (Multimodal Imaging)
神経系イメージング (Neuro Imaging)
機能的神経イメージング(Functional Neuroimaging)
死亡時画像診断(Autopsy imaging)
PET-CT検査 (Positron Emission Tomography Computed Tomography)
放射線療法と画像診断看護 (Radiologic and Imaging Nursing)
ROI (画像診断) (Region of Interest, image diagnosis)
変調伝達関数 (Modulation Transfer Function)

**Figure 2:**
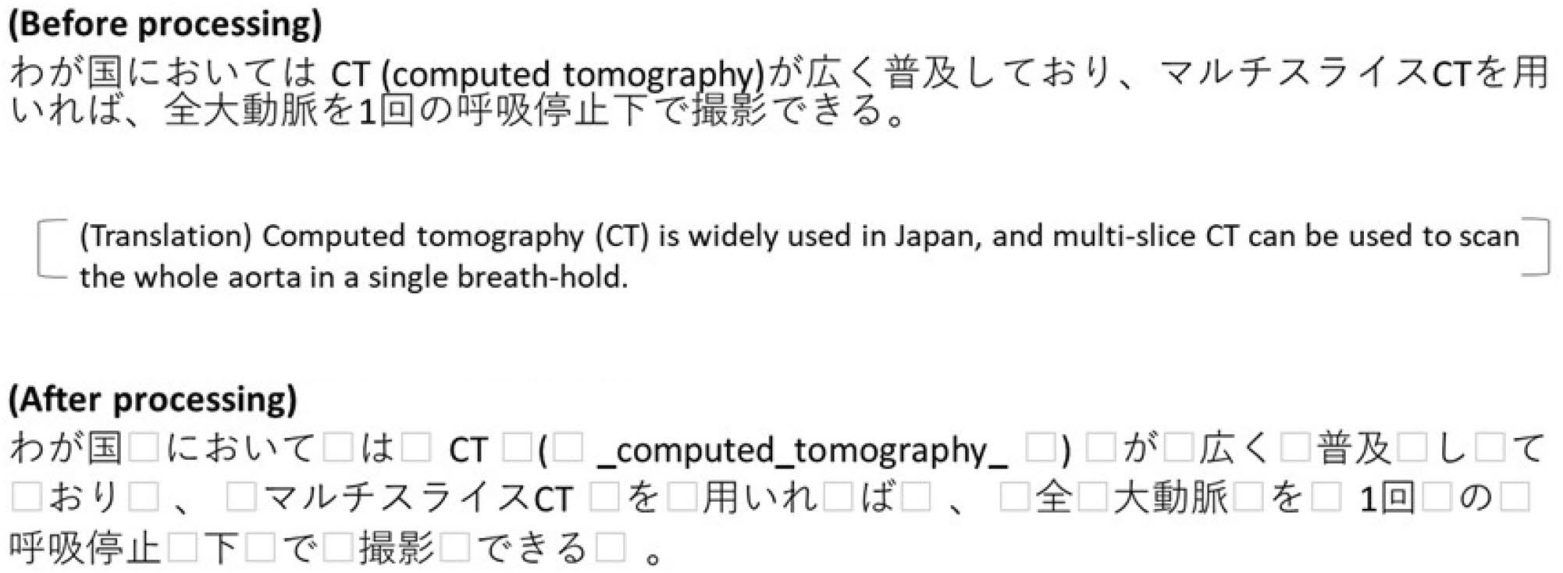
Example of data preprocessing (□: space).

Our preprocessing phase involved several steps. First, we extracted the main text in the abstracts, and inserted spaces ahead of and behind the following symbols, such as brackets, colons, and semicolons. Next, we edited English technical terms consisting more of than 2 words. Specifically, we inserted underbars before, after, and between words appearing in RadLex 4.0^20^ because a word is recognized by the space between words in distributed representations. Finally, we inserted spaces between words using MeCab^21^, which is a morphological analyzer, and the Mecab-ipadic-Neologd dictionary^22^ and the terminology for radiological technology^23,24^. Figure 2 shows an example of the preprocessing.

### Creation of word embedding models

We used two methods: Word2vec^25,26^ and fastText^27^. Word2vec uses the method of learning the context in which a particular word appears using a neural network. There are two architectures to produce a distributed representation of words: CBOW and skip-gram. CBOW is a method of predicting the current word from the surrounding context words; skip-gram is a method of learning the sequence of neighboring words based on the word and its pattern of appearance. fastText is an improved method with n-gram to solve the problem that Word2vec ignores sub-word information and out-of-vocabulary (OOV) words.

The parameters of Word2vec and fastText are shown in Table 2 and were were used to create distributed representations. The Gensim package for Word2vec^28^, and the library of fastText^29^ were used to create training vectors. All experiments for the training models were run on a computer with the Ubuntu 18.04 operating system, Intel Core i7-9700K and 64 GB RAM, with the programming language Python 3.8.3.

**Table 2:** Parameters in Word2vec and fastText.

	Parameters
Architecture	CBOW, Skip-gram
Vector dimensions	100–900
Window size	10
Loss function	Hierarchical softmax
Iteration number	10
Minimum count of words	1
Length of character n-gram(only fastText)	Min: 2, Max: 6

### Synonym set creation

Two radiological technologists collaborated in the development of a synonym set for the evaluation dataset, utilizing terms pertinent to radiology and radiologic technology. Each set in this collection included a Japanese term along with its associated synonyms, which comprised either the corresponding English term and its acronym or the English term alone. This process yielded 1029 sets that became the foundation for this study. To conclude the process, we categorized each set based on fields relevant to the terms. The designated fields include image engineering, physical phenomena, radiation management, equipment, informatics, diagnostic imaging, radiation therapy, and basic medicine (Table 3). For the evaluation of synonym identifications considering expression patterns, we categorized this into seven patterns based on the features of the expression of synonyms, as follows: transliteration variants, different Japanese spellings with the same meaning, Japanese shortened forms, conversion to transliteration, English words, English acronyms, and plural expressions (Table 4).

**Table 3:** Eight fields in radiological technology and their descriptions.

Category	Description	Example	Number
Image engineering	Terms related to imaging engineering such as image processing, image display, and image evaluation	Power spectrum, full width at half maximum, pixel, signal to noise ratio	145
Physical phenomena	Terms representing the interactions between radiation and matter related to radiological examinations, as well as associated materials and phenomena	Radioisotope, echo, free radical, decay	196
Radiation control	Terms related to radiation management such as radiation measuring instruments and dose assessment	Contamination, tracer, yield, cumulative dose	72
Equipment	Terms related to radiological examination and treatment equipment, their parts, and accessories	Grid, linac, flat panel detector, surface coil	168
Informatics	Terms related to information engineering and medical informatics, including statistics and analysis algorithms	Morbidity, sensitivity, decision tree, standardization	89
Diagnostic imaging	Terms related to radiographic imaging examinations, including examination methods and techniques	Coronal plane, intravenous pyelography, cardiothoracic ratio, spin echo	234
Radiation therapy	Terms related to radiation therapy, including treatment techniques	Organs at risk, brachytherapy, preventive irradiation, afterloading	51
Medicine	Terms related to basic medical sciences such as anatomy, physiology, and pathology	Estrogen, immune, cancer, complete response	74

**Table 4:** Expression patterns in synonym sets.

Category		Explanation	Number
Expression patterns in Japanese	Transliteration variants	Pairs of synonyms with different Japanese transliterations	19
	Different Japanese spellings with the same meaning	Pairs of synonym that are different but synonymous. It also includes conversion of character types such as Kanji-hiragana conversion	296
	Japanese shortened forms	Only removing strings when converting one word to the other in synonym pairs	115
	Conversion to transliteration	Pairs of synonyms consisting of an original Japanese word and its transliteration in Japanese	146
	Plural expressions	A synonym set including plural synonyms	453
		Total	1029
English terms		Pairs of synonyms comprising a Japanese original word and its corresponding English terms	1029
English acronyms		Pairs of synonyms of a Japanese original word and English acronyms	140

### Synonym prediction

Subsequently, preferred terms from the synonym sets were input into the distributed representations. The cosine similarities were then calculated, and the top 10 synonym candidates with the highest cosine similarities to the input term were obtained. The formulation of the cosine similarity was presented as follows,

1 $${\text{cosine}} \, \mathrm{ similarity}= \frac{{\text{A}}\cdot {\text{B}}}{\Vert {\text{A}}\Vert \Vert {\text{B}}\Vert }$$

where A is a vector of an input word and B is a vector of a word in a distributed representation.

### Evaluation

The ten synonym candidates procured were juxtaposed against the synonyms delineated in the set. If the orthography of a synonym aligned with that of a candidate, said candidate was adjudged correct. Metrics such as precision, recall, F1-measure, and accuracy were subsequently computed for each word embedding model as follows.

2 $${\text{Precision}} = {\text{TP}}/\left( {{\text{TP}} + {\text{FP}}} \right)$$

3 $${\text{Recall}} = {\text{TP}}/\left( {{\text{TP}} + {\text{FN}}} \right)$$

4 $${\text{F1}} - {\text{score}} = {2} \times {\text{Precision}} \times {\text{Recall}}/\left( {{\text{Precision}} + {\text{Recall}}} \right)$$

5 $${\text{Accuracy}} = \left( {{\text{TP}} + {\text{TN}}} \right)/\left( {{\text{TP}} + {\text{FP}} + {\text{FN}} + {\text{TN}}} \right)$$

Where TP stands for true positive and refers to situations wherein a word is correctly identified as a synonym. FP denotes false positive and indicates instances wherein a word is erroneously identified as a synonym when, in fact, it is not. FN signifies false negative and pertains to situations in which a word is inaccurately identified as not being a synonym by the models. TN refers to true negative and represents cases wherein a word is correctly identified as not being a synonym. However, TN was set to 0 in this study due to the stipulation that only synonymous candidate words would be output in this task.

For each model, cumulative accuracies across various ranks were derived by consolidating individual prediction accuracies within the model using the formula in Eq. (5). Moreover, to gauge the accuracy of synonym identification contingent upon expression patterns, we computed the metrics of precision, recall, F1-score, and accuracy over eight specific fields in radiological technology and different expression patterns in the synonym sets. In the analysis of English terms and abbreviations, the output was also assessed based on words consisting solely of alphabetical characters.

### Ethical approval

This article does not include any studies with human participants or animals that were performed by any of the authors.

## Results

### Comparison among models

In comparing the evaluation indices of synonym predictions across each word embedding model, the model that achieved the highest precision, recall, F1-score and accuracy was fastText with CBOW with 300 vector dimensions registering scores of 0.5567, 0.8872, 0.6841, 0.5199, respectively (Table 5). In all evaluation indices, the observed values indicated that accuracies in fastText consistently outperformed those of Word2vec. The observed trends for CBOW were superior to those of skip-gram. However, in fastText with CBOW, the 100-dimensional representation yielded the lowest performance. There was a general trend of improved performance with increasing dimensions in both fastText with skip-gram and Word2vec with CBOW. Conversely, in Word2vec with skip-gram, performance declined as dimensionality decreased.

**Table 5:** Cumulative ratio of synonym prediction in each word embedding model. (FT: fastText, W2V:Word2vec, SKIP:skip-gram, number: vector dimensions).

Model	Precition	Recall	F1-score	Accuracy
FT_CBOW_100	0.5286	0.8819	0.6610	0.4937
FT_CBOW_200	0.5557	0.8870	0.6833	0.5190
FT_CBOW_300	**0.5567**	**0.8872**	**0.6841**	**0.5199**
FT_CBOW_400	0.5515	0.8863	0.6799	0.5151
FT_CBOW_500	0.5484	0.8857	0.6774	0.5121
FT_CBOW_600	0.5432	0.8847	0.6731	0.5073
FT_CBOW_700	0.5359	0.8834	0.6671	0.5005
FT_CBOW_800	0.5369	0.8836	0.6680	0.5015
FT_CBOW_900	0.5276	0.8817	0.6602	0.4927
FT_SKIP_100	0.4381	0.8609	0.5807	0.4091
FT_SKIP_200	0.4984	0.8757	0.6353	0.4655
FT_SKIP_300	0.4984	0.8757	0.6353	0.4655
FT_SKIP_400	0.5213	0.8805	0.6549	0.4869
FT_SKIP_500	0.5130	0.8788	0.6478	0.4791
FT_SKIP_600	0.5317	0.8826	0.6636	0.4966
FT_SKIP_700	0.5369	0.8836	0.6680	0.5015
FT_SKIP_800	0.5297	0.8821	0.6619	0.4947
FT_SKIP_900	0.5317	0.8826	0.6636	0.4966
W2V_CBOW_100	0.3101	0.8142	0.4491	0.2896
W2V_CBOW_200	0.3757	0.8415	0.5194	0.3508
W2V_CBOW_300	0.3809	0.8433	0.5247	0.3557
W2V_CBOW_400	0.3954	0.8482	0.5394	0.3693
W2V_CBOW_500	0.3985	0.8492	0.5425	0.3722
W2V_CBOW_600	0.3913	0.8468	0.5352	0.3654
W2V_CBOW_700	0.3965	0.8486	0.5404	0.3703
W2V_CBOW_800	0.4089	0.8525	0.5527	0.3819
W2V_CBOW_900	0.3944	0.8479	0.5384	0.3683
W2V_SKIP_100	0.3195	0.8187	0.4596	0.2983
W2V_SKIP_200	0.3278	0.8225	0.4688	0.3061
W2V_SKIP_300	0.3195	0.8187	0.4596	0.2983
W2V_SKIP_400	0.3195	0.8187	0.4596	0.2983
W2V_SKIP_500	0.3070	0.8127	0.4456	0.2867
W2V_SKIP_600	0.2955	0.8068	0.4326	0.2760
W2V_SKIP_700	0.2872	0.8023	0.4230	0.2682
W2V_SKIP_800	0.2893	0.8035	0.4254	0.2702
W2V_SKIP_900	0.2851	0.8012	0.4206	0.2663

The underlined (bold) numbers show the highest cumulated accuracy in the different models.

**Figure 3:**
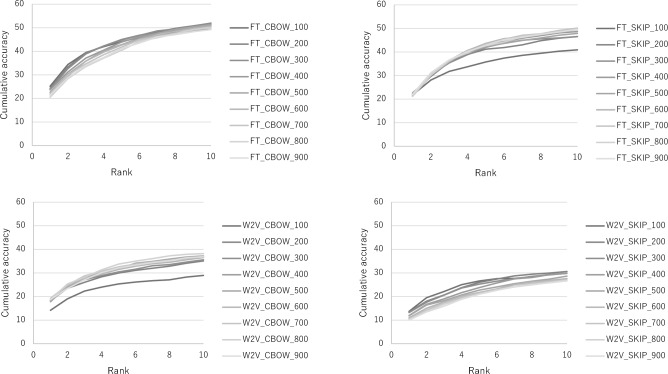
Evaluation of each model (top left: Precision, top right: Recall, bottom left: F1-score, bottom right: Accuracy).

Figure 3 illustrates the cumulative probabilities observed in each model. In the context of the Word2vec with the CBOW model, the average variation in cumulative ratios for the foremost 1–10 words was observed to be approximately 14.8% to 19.5%. In contrast, the fastText with CBOW model exhibited a range of about 24.1% to 29.5%, and the fastText with skip-gram model displayed a span of 19.0% to 28.2%. "It is noteworthy that in the fastText model, there was a marked enhancement in cumulative accuracy when incorporating terms that appeared in lower ranks. Table 6 shows examples of synonym candidates (Top-10) using the most accurate model in each architecture.

**Table 6:** Examples of the Top-10 synonym candidates in the models with the greatest accuracy.

Input term	model	1st candidate	2nd candidate	3rd candidate	4th candidate	5th candidate	6th candidate	7th candidate	8th candidate	9th candidate	10th candidate
アーキテクチャ(architecture)	Word2vec with CBOW and 800 dimension	Fukushima	GPU	ネオコグニトロン	CHROMagar	HardDisk	アルゴリズム	タブレット端末	convolutional	ニューラルネットワーク	手法
Synonym (transliteration variants); アーキテクチャー(architecture)				(Neocognitron)			(algorithm)	(tablet device)		(neural network)	(method)
	Word2vec with skip-gram 200 dimension	Fukushima	ネオコグニトロン	CUDA	CBMIR	ランダムフォレスト	Programming	入力支援	Keras	convolutional	Framework
			(Neocognitron)			(random forest)		(input support)			
	fastText with CBOW and 300 dimension	認知アーキテクチャ	**アーキテクチャー**	システムアーキテクチャ	マイクロアーキテクチャ	アーキテクト	ディープニューラルネットワーク	ストラクチャ	ブレインネットワークインタフェース	医用画像処理システム	ニューラルネットワーク
		(cognitive architecture)	**(architecture)**	(system architecture)	(microarchitecture)	(architect)	(deep neural network)	(structure)	(brain network interface)	(medical image processing system)	(neural network)
	fastText with skip-gram and 800 dimension	認知アーキテクチャ	**アーキテクチャー**	システムアーキテクチャ	マイクロアーキテクチャ	アーキテクト	ネオコグニトロン	ディープコンボリューションニューラルネット	ディープニューラルネットワーク	ニューラルネットワーク	ブレイン・ネットワークインタフェース
		(cognitive architecture)	**(architecture)**	(system architecture)	(microarchitecture)	(architect)	(Neocognitron)	(deep convolutional neural network)	(deep neural network)	(neural network)	(brain network interface)
コントラスト改善度(contrast improvement factor)	Word2vec with CBOW and 800 dimension	選択度	露出倍数	ビット	Nkx	グリッド比	散乱線含有率	) ~	ウシアブ	陽性尤度比	+ ~
Synonym (Japanese different spelling with the same meaning); コントラスト改善比(contrast improvement ratio)		(selectivity)	(exposure magnifying factor)	(bit)		(grid ratio)	(content rate of scattered radiation)		(horsefly)	(positive likelihood ratio)	
	Word2vec with skip-gram 200 dimension	選択度	露出倍数	全X線透過率	散乱線含有率	モルト	集束距離	THX	180 mA	クロスグリッド	GIPAN
		(selectivity)	(exposure magnifying factor)	(total transmission of X-rays)	(content rate of scattered radiation)	(malt)	(focusing distance)			(cross grid)	
	fastText with CBOW and 300 dimension	**コントラスト改善比(contrast improvement ratio)**	コントラストディテイル	コントラストピークタイム	コントラスト	コントラスト比	コントラスト	コントラストハーモニックパワーフロー	低コントラスト解像度	コントラスト雑音比	コントラストエンハンスメント
			(contrast detail)	(contrast peak time)	(contrast)	(contrast ratio)	(contrast)	(contrast harmonic power flow)	(low-contrast resolution)	(contrast to noise ratio)	(contrast enhancement)
	fastText with skip-gram and 800 dimension	**コントラスト改善比(contrast improvement ratio)**	選択度	露出倍数	コントラストピークタイム	コントラストディテイル	全X線透過率	コントラスト	コントラスト比	散乱線含有率	低コントラスト分解能
			(selectivity)	(exposure magnifying factor)	(contrast peak time)	(contrast detail)	(total transmission of X-rays)	(contrast)	(contrast ratio)	(content rate of scattered radiation)	(low-contrast resolution)
解像度(resolution)	Word2vec with CBOW and 800 dimension	**分解能**	空間分解能	精細	**解像力**	品位	時間分解能	フレームレート	侵達(penetration)	速多^a^	倍率
Synonym (Plural expressions); 解像力(resolution power), 分解能(resolution)		**(resolution)**	(spatial resolution)	(high definition)	**(resolution power)**	(quality)	(time resolution)	(frame rate)			(magnification)
	Word2vec with skip-gram 200 dimension	**分解能**	空間分解能	精細	**解像力**	時間分解能	高画質	高速	画質	高速化	コントラスト
		**(resolution)**	(spatial resolution)	(high definition)	**(resolution power)**	(time resolution)	(high image quality)	(high speed)	(image quality)	(speeding up)	(contrast)
	fastText with CBOW and 300 dimension	**分解能**	水平解像度	精細	空間分解能	垂直分解能	位置分解能	**解像力**	固有分解能	総合分解能	システム分解能
		**(resolution)**	(horizontal resolution)	(high definition)	(spatial resolution)	(vertical resolution)	(position resolution)	**(resolution power)**	(intrinsic resolution)	(system resolution)	(system resolution)
	fastText with skip-gram and 800 dimension	**分解能**	空間分解能	水平解像度	**解像力**	精細	時間分解能	垂直解像度	システム分解能	高コントラスト解像度	超解像
		**(resolution)**	(spatial resolution)	(horizontal resolution)	**(resolution power)**	(high definition)	(time resolution)	(vertical resolution)	(system resolution)	(high contrast resolution)	(super-resolution)
ラジオアイソトープ(radioisotope)	Word2vec with CBOW and 800 dimension	**radioisotope**	ベノグラフィ	**放射性同位元素**	isotope	スキップラミネクトミー	塩化タリウム	ARG	SLNB	テクネチウム-99 m	registratin
Synonym (Plural expressions): 放射性核種(radionuclides), 放射性同位元素(radioisotope), radioactive isotope, etc			(venography)	**(radioisotope)**		(skip laminectomy)	(thallium chloride)			(technetium 99 m)	
	Word2vec with skip-gram 200 dimension	**放射性同位元素**	radioisotope	アイソトープ	トレーサ	リンフォシンチグラム	リンパシンチグラフィー	パテント	トレーサー	パテントブルーバイオレット	硫化アンチモン
		**(radioisotope)**		(isotope)	(tracer)	(lymphoscintigram)	(lymphatic scintigraphy)	(patent)	(tracer)	(patent blue violet)	(antimony sulfide)
	fastText with CBOW and 300 dimension	ラジオアイソトープ検査	ライジオアイソトープ	ラジオアイオソトープシンチグラフィ	アイソトープ	リンフォシンチグラフィ	**放射性同位体**	リンフォシンチグラム	リンパシンチグラフィー	アンソトープ	ダイナミックリンパシンチグラフィー
		(radio isotope examination)	(radioisotope / erratum)	(radioisotope scintigraphy)	(isotope)	(lymphoscintigraphy)	**(radioisotope)**	(lymphoscintigram)	(lymphatic scintigraphy)		(dynamic lymphatic scintigraphy)
	fastText with skip-gram and 800 dimension	ライジオアイソトープ	ラジオアイソトープ検査	ラジオアイオソトープシンチグラフィ	アイソトープ	**radioisotope**	**Radioisotope**	センチネルリンパシンチグラフ	**放射性同位元素**	センチネルリンパシンチ	リンフォシンチグラフィー
		(radioisotope)	(radio isotope examination)	(radioisotope scintigraphy)	(isotope)			(Sentinel lymph node scintigraph)	**(radioisotope)**	(Sentinel lymph node scintigraphy)	(lymphatic scintigraphy)
直接X線撮影(direct X-ray radiography)	Word2vec with CBOW and 800 dimension	間接X線撮影	**直接撮影**	間接撮影	X線検査	集団検診	Thoravision	X線撮影	9664	X線撮影法	Veraview
Synonym (Plural expressions); 直接撮影(direct radiography), 直接撮影法(direct radiography method)		(indirect X-ray radiography)	**(direct radiography)**	(indirect radiography)	(X-ray examination)	(mass health screening)		(x-ray radiography)		(x-ray radiography method)	
	Word2vec with skip-gram 200 dimension	間接X線撮影	間接撮影	**直接撮影**	3108	集団検診	82,596	1,012,976	21,979	茨城県総合健診協会	2,521,546
		(indirect X-ray radiography)	(indirect radiography)	**(direct radiography)**		(mass health screening)				(Ibaraki Health Service Association)	
	fastText with CBOW and 300 dimension	間接X線撮影	直接X線像	間接X線撮影法	接線撮影	立体X線撮影	X線撮影	**直接撮影**	デジタルX線撮影	**直接撮影法**	口外撮影
		(indirect X-ray radiography)	(direct X-ray image)	(indirect X-ray radiography method)	(tangential radiography)	(stereo X-ray radiography)	(x-ray radiography)	**(direct radiography)**	(digital x-ray radiographyy)	**(direct radiography)**	(extraoral radiography)
	fastText with skip-gram and 800 dimension	直接X線像	間接X線撮影	間接X線撮影法	**直接撮影**	間接撮影	間接撮影検査	**直接撮影法**	立体X線撮影	接線撮影	集団検診
		(direct X-ray image)	(indirect X-ray radiography)	(indirect X-ray radiography method)	**(direct radiography)**	(indirect radiography)	(indirect radiography)	**(direct radiography method)**	(stereo X-ray radiography)	(tangential radiography)	(mass health screening)
高速フーリエ変換 (fast Fourier transform)	Word2vec with CBOW and 800 dimension	**FFT**	フーリエ変換	最小二乗法	パワースペクトル	独立成分分析	級数	計算ソフト	自己相関	周波数成分	送波
Synonym (Japanese words and English acronyms): FFT			(fast fourier transform)	(least squares method)	(power spectrum)	(Independent component analysis)	(series)	(calculation software)	(autocorrelation)	(frequency component)	(wave transmission)
	Word2vec with skip-gram 200 dimension	**FFT**	反射係数	Cepstrum	位相	周波数	ヒルベルト変換	ひまし油	検波	スーパーインポーザ	同期信号
			(reflection coefficient)		(phase)	(frequency)	(Hilbert transformation)	(castor oil)	(modulation)	(superimposer)	(synchronizing signal)
	fastText with CBOW and 300 dimension	離散フーリエ変換	短時間フーリエ変換	フーリエ変換	フーリエ級数	フーリエー	余弦変換	パワースペクトル	波形	画像変換	高速Fourier変換
		(discrete Fourier transform)	(Short-time Fourier transform)	(Fourier transform)	(Fourier series)	(Fourier)	(cosine transform)	(power spectrum)	(waveform)	(image conversion)	(fast fourier transform)
	fastText with skip-gram and 800 dimension	短時間フーリエ変換	離散フーリエ変換	フーリエ変換	**FFT**	周波数	ドプラースペクトル	パワースペクトル	周波数スペクトル	逆変換	フーリエー
		(Short-time Fourier transform)	(discrete Fourier transform)	(Fourier transform)		(frequency)	(doppler spectrum)	(power spectrum)	(frequency spectrum)	(inverse transformation)	(Fourier)

^a^Misdivision by the morphological analysis.

Underlined bold terms are synonyms in the baseline.

### Analysis for the synonym expression patterns in Japanese

Table 7 presents the results for synonym expression patterns in Japanese. In the “different Japanese spellings with the same meaning,” almost all fastText models demonstrated superior performance over word2vec across all indices. The fastText model employing CBOW at 400 dimensions reported precision, recall, F1-score, and accuracy values of 0.5365, 0.6649, 0.5938, and 0.4222, respectively.

**Table 7:** Results for synonym expression patterns in Japanese.(FT: fastText, W2V:Word2vec, SKIP:skip-gram, number: vector dimensions).

Model	Different Japanese spellings with the same meaning				Japanese shortened forms				Conversion to transliteration				Transliteration variants				Plural expressions			
	Precition	Recall	F1-score	Accuracy	Precition	Recall	F1-score	Accuracy	Precition	Recall	F1-score	Accuracy	Precition	Recall	F1-score	Accuracy	Precition	Recall	F1-score	Accuracy
FT_CBOW_100	0.4721	0.6358	0.5419	0.3716	0.8269	0.8866	0.8557	0.7478	0.1885	0.4894	0.2722	0.1575	0.6667	0.9231	0.7742	0.6316	0.6472	0.9172	0.7589	0.6115
FT_CBOW_200	0.5236	0.6595	0.5837	0.4122	0.8462	0.8889	0.8670	0.7652	0.2049	0.5102	0.2924	0.1712	0.7222	0.9286	0.8125	0.6842	0.6682	0.9196	0.7740	0.6313
FT_CBOW_300	0.5322	0.6631	0.5905	0.4189	0.8654	0.8911	0.8780	0.7826	0.1721	0.4667	0.2515	0.1438	0.7222	0.9286	0.8125	0.6842	**0.6706**	**0.9199**	**0.7757**	**0.6336**
FT_CBOW_400	**0.5365**	**0.6649**	**0.5938**	**0.4223**	0.8846	0.8932	0.8889	0.8000	0.1885	0.4894	0.2722	0.1575	0.7222	0.9286	0.8125	0.6842	0.6472	0.9172	0.7589	0.6115
FT_CBOW_500	0.5279	0.6613	0.5871	0.4155	0.8750	0.8922	0.8835	0.7913	0.1721	0.4667	0.2515	0.1438	0.7222	0.9286	0.8125	0.6842	0.6519	0.9178	0.7623	0.6159
FT_CBOW_600	0.5279	0.6613	0.5871	0.4155	0.8942	0.8942	0.8942	0.8087	0.1557	0.4419	0.2303	0.1301	0.7778	0.9333	0.8485	0.7368	0.6379	0.9161	0.7521	0.6026
FT_CBOW_700	0.5150	0.6557	0.5769	0.4054	0.8750	0.8922	0.8835	0.7913	0.1557	0.4419	0.2303	0.1301	0.7222	0.9286	0.8125	0.6842	0.6355	0.9158	0.7503	0.6004
FT_CBOW_800	0.5150	0.6557	0.5769	0.4054	0.8942	0.8942	0.8942	0.8087	0.1557	0.4419	0.2303	0.1301	0.7778	0.9333	0.8485	0.7368	0.6308	0.9153	0.7469	0.5960
FT_CBOW_900	0.4721	0.6358	0.5419	0.3716	**0.9038**	**0.8952**	**0.8995**	**0.8174**	0.1393	0.4146	0.2086	0.1164	0.7222	0.9286	0.8125	0.6842	0.6379	0.9161	0.7521	0.6026
FT_SKIP_100	0.3562	0.5685	0.4380	0.2804	0.5962	0.8493	0.7006	0.5391	0.1967	0.5000	0.2824	0.1644	0.7778	0.9333	0.8485	0.7368	0.5561	0.9049	0.6889	0.5254
FT_SKIP_200	0.4163	0.6063	0.4936	0.3277	0.6923	0.8675	0.7701	0.6261	0.2377	0.5472	0.3314	0.1986	**0.8333**	**0.9375**	**0.8824**	**0.7895**	0.6215	0.9141	0.7399	0.5872
FT_SKIP_300	0.4292	0.6135	0.5051	0.3378	0.7115	0.8706	0.7831	0.6435	0.2049	0.5102	0.2924	0.1712	0.7778	0.9333	0.8485	0.7368	0.6215	0.9141	0.7399	0.5872
FT_SKIP_400	0.4764	0.6379	0.5455	0.3750	0.7308	0.8736	0.7958	0.6609	0.2213	0.5294	0.3121	0.1849	**0.8333**	**0.9375**	**0.8824**	**0.7895**	0.6355	0.9158	0.7503	0.6004
FT_SKIP_500	0.4807	0.6400	0.5490	0.3784	0.7692	0.8791	0.8205	0.6957	0.1967	0.5000	0.2824	0.1644	0.7778	0.9333	0.8485	0.7368	0.6145	0.9132	0.7346	0.5806
FT_SKIP_600	0.4850	0.6420	0.5526	0.3818	0.7692	0.8791	0.8205	0.6957	0.2213	0.5294	0.3121	0.1849	0.7778	0.9333	0.8485	0.7368	0.6472	0.9172	0.7589	0.6115
FT_SKIP_700	0.4893	0.6441	0.5561	0.3851	0.8269	0.8866	0.8557	0.7478	0.2213	0.5294	0.3121	0.1849	0.7222	0.9286	0.8125	0.6842	0.6449	0.9169	0.7572	0.6093
FT_SKIP_800	0.4807	0.6400	0.5490	0.3784	0.7981	0.8830	0.8384	0.7217	0.2213	0.5294	0.3121	0.1849	0.7222	0.9286	0.8125	0.6842	0.6402	0.9164	0.7538	0.6049
FT_SKIP_900	0.4807	0.6400	0.5490	0.3784	0.8173	0.8854	0.8500	0.7391	0.2295	0.5385	0.3218	0.1918	0.7222	0.9286	0.8125	0.6842	0.6379	0.9161	0.7521	0.6026
W2V_CBOW_100	0.2661	0.4960	0.3464	0.2095	0.2308	0.6857	0.3453	0.2087	0.2213	0.5294	0.3121	0.1849	0.6111	0.9167	0.7333	0.5789	0.4065	0.8744	0.5550	0.3841
W2V_CBOW_200	0.3348	0.5532	0.4171	0.2635	0.3077	0.7442	0.4354	0.2783	0.2951	0.6000	0.3956	0.2466	0.6111	0.9167	0.7333	0.5789	0.4766	0.8908	0.6210	0.4503
W2V_CBOW_300	0.3691	0.5772	0.4503	0.2905	0.2885	0.7317	0.4138	0.2609	0.3033	0.6066	0.4044	0.2534	0.5556	0.9091	0.6897	0.5263	0.4743	0.8904	0.6189	0.4481
W2V_CBOW_400	0.3948	0.5935	0.4742	0.3108	0.2885	0.7317	0.4138	0.2609	**0.3279**	**0.6250**	**0.4301**	**0.2740**	0.6667	0.9231	0.7742	0.6316	0.4813	0.8918	0.6252	0.4547
W2V_CBOW_500	0.3734	0.5800	0.4543	0.2939	0.3077	0.7442	0.4354	0.2783	0.2951	0.6000	0.3956	0.2466	0.6667	0.9231	0.7742	0.6316	0.5047	0.8963	0.6457	0.4768
W2V_CBOW_600	0.3605	0.5714	0.4421	0.2838	0.3077	0.7442	0.4354	0.2783	0.3197	0.6190	0.4216	0.2671	0.5556	0.9091	0.6897	0.5263	0.4930	0.8941	0.6355	0.4658
W2V_CBOW_700	0.3777	0.5828	0.4583	0.2973	0.3173	0.7500	0.4459	0.2870	0.3115	0.6129	0.4130	0.2603	0.5556	0.9091	0.6897	0.5263	0.4953	0.8945	0.6376	0.4680
W2V_CBOW_800	0.3991	0.5962	0.4781	0.3142	0.2981	0.7381	0.4247	0.2696	0.3197	0.6190	0.4216	0.2671	0.7222	0.9286	0.8125	0.6842	0.5070	0.8967	0.6478	0.4790
W2V_CBOW_900	0.3777	0.5828	0.4583	0.2973	0.2692	0.7179	0.3916	0.2435	0.2951	0.6000	0.3956	0.2466	0.5556	0.9091	0.6897	0.5263	0.5070	0.8967	0.6478	0.4790
W2V_SKIP_100	0.2575	0.4878	0.3371	0.2027	0.2692	0.7179	0.3916	0.2435	0.2295	0.5385	0.3218	0.1918	0.4444	0.8889	0.5926	0.4211	0.4276	0.8798	0.5755	0.4040
W2V_SKIP_200	0.2833	0.5116	0.3646	0.2230	0.2788	0.7250	0.4028	0.2522	0.2459	0.5556	0.3409	0.2055	0.4444	0.8889	0.5926	0.4211	0.4252	0.8792	0.5732	0.4018
W2V_SKIP_300	0.2876	0.5154	0.3691	0.2264	0.2885	0.7317	0.4138	0.2609	0.2541	0.5636	0.3503	0.2123	0.4444	0.8889	0.5926	0.4211	0.3995	0.8724	0.5481	0.3775
W2V_SKIP_400	0.3047	0.5299	0.3869	0.2399	0.2885	0.7317	0.4138	0.2609	0.2459	0.5556	0.3409	0.2055	0.4444	0.8889	0.5926	0.4211	0.3925	0.8705	0.5411	0.3709
W2V_SKIP_500	0.2961	0.5227	0.3781	0.2331	0.2788	0.7250	0.4028	0.2522	0.2213	0.5294	0.3121	0.1849	0.4444	0.8889	0.5926	0.4211	0.3785	0.8663	0.5268	0.3576
W2V_SKIP_600	0.2918	0.5191	0.3736	0.2297	0.2692	0.7179	0.3916	0.2435	0.2295	0.5385	0.3218	0.1918	0.3333	0.8571	0.4800	0.3158	0.3598	0.8603	0.5074	0.3400
W2V_SKIP_700	0.2618	0.4919	0.3417	0.2061	0.2692	0.7179	0.3916	0.2435	0.2459	0.5556	0.3409	0.2055	0.3889	0.8750	0.5385	0.3684	0.3505	0.8571	0.4975	0.3311
W2V_SKIP_800	0.2918	0.5191	0.3736	0.2297	0.2981	0.7381	0.4247	0.2696	0.2049	0.5102	0.2924	0.1712	0.4444	0.8889	0.5926	0.4211	0.3411	0.8538	0.4875	0.3223
W2V_SKIP_900	0.2704	0.5000	0.3510	0.2128	0.2788	0.7250	0.4028	0.2522	0.2049	0.5102	0.2924	0.1712	0.3889	0.8750	0.5385	0.3684	0.3505	0.8571	0.4975	0.3311

The underlined (bold) numbers show the highest cumulated accuracy in the different models.

In the “Japanese shortened forms,” the indices for fastText notably surpassed those of word2vec. The optimal performance was observed in fastText with CBOW at 900 dimensions, yielding precision, recall, F1-score, and accuracy values of 0.9038, 0.8952, 0.9000, and 0.8174, respectively. Furthermore, there was a discernible enhancement in performance as the dimension number increased.

In “conversion to transliteration,” word2vec models with CBOW outshined other models in all indices. Specifically, the model at 400 dimensions achieved a precision of 0.328, a recall of 0.625, an F1-score of 0.430, and an accuracy of 0.274.

In “transliteration variants,” fastText models consistently excelled over those of word2vec. The skip-gram approach at both 200 and 400 dimensions exhibited standout performance, registering precision, recall, F1-score, and accuracy values of 0.833, 0.935, 0.882, and 0.789, respectively.

**Figure 4:**
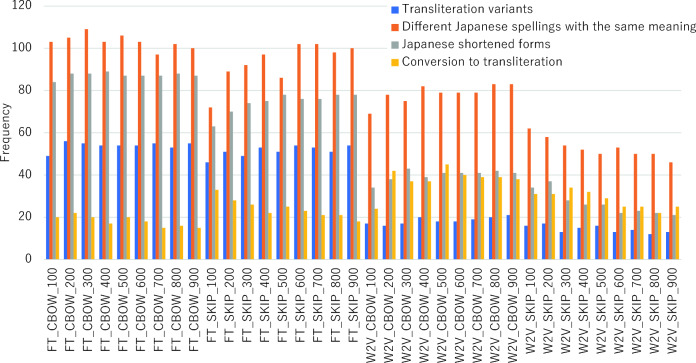
Frequency of synonym expression patterns detected in multiple expressions (FT: fastText, W2V:Word2vec, SKIP:skip-gram, number: vector dimensions).

In the category of “plural expressions,” fastText models significantly outperformed Word2vec models, particularly excelling in precision, recall, and overall accuracy. The fastText model employing a 300-dimensional CBOW architecture emerged as the most effective. Figure 4 presents the distribution of the most prominent synonym expression patterns for plural expressions, as analyzed across various models. For categories such as “transliteration variants,” “different Japanese spellings with the same meaning,” and “Japanese shortened forms,” optimal performance was achieved within the 200 to 400 dimensional range using fastText with CBOW. In contrast, other notation patterns showed a tendency towards Word2vec, with “conversion to transliteration” peaking with a 500-dimensional CBOW model.

### Analysis for the synonym expression patterns in English terms and abbreviations

Table 8 shows the results for English terms and abbreviations. In “Japanese and English terms,” all indices were poor for all models. The best F1-score and accuracy were 0.1836 and 0.1011 for word2vec with CBOW and 800 dimensions. In Output alphabetic-only words, the values of all indicators improved, and the improvement was particularly pronounced in fastText. The best model was fastText with skipgram at 800 and 900 dimensions, with precision, recall, F1-score and accuracy of 0.3253, 0.3718, 0.3470 and 0.2099, respectively. The rate of increase ranged from 0.15 to 0.25 for all indices.

**Table 8:** Results for synonym expression patterns in English terms and acronyms.(FT: fastText, W2V:Word2vec, SKIP:skip-gram, number: vector dimensions).

Model	English terms				English acronyms				English terms (Output alphabetic-only words)				English acronyms (Output alphabetic-only words)			
	Precition	Recall	F1-score	Accuracy	Precition	Recall	F1	Accuracy	Precition	Recall	F1-score	Accuracy	Precition	Recall	F1-score	Accuracy
FT_CBOW_100	0.0813	0.1289	0.0997	0.0525	0.3143	1.0000	0.4783	0.3143	0.2395	0.3034	0.2677	0.1545	0.4214	1.0000	0.5930	0.4214
FT_CBOW_200	0.0708	0.1141	0.0874	0.0457	0.3429	1.0000	0.5106	0.3429	0.2575	0.3190	0.2850	0.1662	0.5071	1.0000	0.6730	0.5071
FT_CBOW_300	0.0678	0.1098	0.0838	0.0437	0.3286	1.0000	0.4946	0.3286	0.2801	0.3376	0.3062	0.1808	0.5143	1.0000	0.6792	0.5143
FT_CBOW_400	0.0633	0.1032	0.0784	0.0408	0.3000	1.0000	0.4615	0.3000	0.2786	0.3364	0.3048	0.1798	**0.5500**	**1.0000**	**0.7097**	**0.5500**
FT_CBOW_500	0.0602	0.0988	0.0748	0.0389	0.2857	1.0000	0.4444	0.2857	0.2741	0.3327	0.3006	0.1769	0.5357	1.0000	0.6977	0.5357
FT_CBOW_600	0.0512	0.0852	0.0640	0.0330	0.2571	1.0000	0.4091	0.2571	0.2651	0.3253	0.2921	0.1710	0.5143	1.0000	0.6792	0.5143
FT_CBOW_700	0.0452	0.0759	0.0567	0.0292	0.2500	1.0000	0.4000	0.2500	0.2937	0.3482	0.3186	0.1895	0.4929	1.0000	0.6603	0.4929
FT_CBOW_800	0.0407	0.0689	0.0511	0.0262	0.2286	1.0000	0.3721	0.2286	0.2741	0.3327	0.3006	0.1769	0.5357	1.0000	0.6977	0.5357
FT_CBOW_900	0.0346	0.0593	0.0437	0.0224	0.2429	1.0000	0.3908	0.2429	0.2696	0.3290	0.2964	0.1740	0.5214	1.0000	0.6854	0.5214
FT_SKIP_100	0.1009	0.1551	0.1223	0.0651	0.3857	1.0000	0.5567	0.3857	0.2395	0.3034	0.2677	0.1545	0.4214	1.0000	0.5930	0.4214
FT_SKIP_200	0.1250	0.1853	0.1493	0.0807	0.4357	1.0000	0.6070	0.4357	0.2907	0.3459	0.3159	0.1876	0.5143	1.0000	0.6792	0.5143
FT_SKIP_300	0.1099	0.1667	0.1325	0.0709	0.4357	1.0000	0.6070	0.4357	0.3133	0.3630	0.3363	0.2021	0.5286	1.0000	0.6916	0.5286
FT_SKIP_400	0.1054	0.1609	0.1274	0.0680	**0.4643**	**1.0000**	**0.6341**	**0.4643**	0.3178	0.3663	0.3403	0.2051	0.5429	1.0000	0.7037	0.5429
FT_SKIP_500	0.0994	0.1531	0.1205	0.0641	0.4357	1.0000	0.6070	0.4357	0.3238	0.3707	0.3457	0.2089	**0.5500**	**1.0000**	**0.7097**	**0.5500**
FT_SKIP_600	0.1054	0.1609	0.1274	0.0680	0.4357	1.0000	0.6070	0.4357	0.3102	0.3608	0.3336	0.2002	**0.5500**	**1.0000**	**0.7097**	**0.5500**
FT_SKIP_700	0.0949	0.1472	0.1154	0.0612	0.4429	1.0000	0.6139	0.4429	0.2997	0.3528	0.3241	0.1934	0.5429	1.0000	0.7037	0.5429
FT_SKIP_800	0.0904	0.1412	0.1102	0.0583	0.4286	1.0000	0.6000	0.4286	**0.3253**	**0.3718**	**0.3470**	**0.2099**	0.5429	1.0000	0.7037	0.5429
FT_SKIP_900	0.0828	0.1310	0.1015	0.0534	0.4214	1.0000	0.5930	0.4214	**0.3253**	**0.3718**	**0.3470**	**0.2099**	**0.5500**	**1.0000**	**0.7097**	**0.5500**
W2V_CBOW_100	0.1069	0.1628	0.1291	0.0690	0.3429	1.0000	0.5106	0.3429	0.1822	0.2490	0.2104	0.1176	0.4643	1.0000	0.6341	0.4643
W2V_CBOW_200	0.1280	0.1889	0.1526	0.0826	0.3857	1.0000	0.5567	0.3857	0.2289	0.2940	0.2574	0.1477	0.4214	1.0000	0.5930	0.4214
W2V_CBOW_300	0.1325	0.1943	0.1576	0.0855	0.4000	1.0000	0.5714	0.4000	0.2244	0.2899	0.2530	0.1448	0.4786	1.0000	0.6473	0.4786
W2V_CBOW_400	0.1401	0.2031	0.1658	0.0904	0.4143	1.0000	0.5859	0.4143	0.2244	0.2899	0.2530	0.1448	0.4786	1.0000	0.6473	0.4786
W2V_CBOW_500	0.1461	0.2100	0.1723	0.0943	0.4000	1.0000	0.5714	0.4000	0.2259	0.2913	0.2545	0.1458	0.4643	1.0000	0.6341	0.4643
W2V_CBOW_600	0.1461	0.2100	0.1723	0.0943	0.4071	1.0000	0.5787	0.4071	0.2244	0.2899	0.2530	0.1448	0.4500	1.0000	0.6207	0.4500
W2V_CBOW_700	0.1416	0.2048	0.1674	0.0914	0.3929	1.0000	0.5641	0.3929	0.2274	0.2926	0.2559	0.1467	0.4500	1.0000	0.6207	0.4500
W2V_CBOW_800	**0.1566**	**0.2217**	**0.1836**	**0.1011**	0.3857	1.0000	0.5567	0.3857	0.2334	0.2981	0.2618	0.1506	0.4571	1.0000	0.6275	0.4571
W2V_CBOW_900	0.1491	0.2134	0.1755	0.0962	0.3786	1.0000	0.5492	0.3786	0.2395	0.3034	0.2677	0.1545	0.4286	1.0000	0.6000	0.4286
W2V_SKIP_100	0.1340	0.1960	0.1592	0.0865	0.3714	1.0000	0.5417	0.3714	0.2093	0.2758	0.2380	0.1351	0.4429	1.0000	0.6139	0.4429
W2V_SKIP_200	0.1295	0.1907	0.1543	0.0836	0.3929	1.0000	0.5641	0.3929	0.2214	0.2871	0.2500	0.1429	0.4500	1.0000	0.6207	0.4500
W2V_SKIP_300	0.1355	0.1978	0.1609	0.0875	0.3929	1.0000	0.5641	0.3929	0.2289	0.2940	0.2574	0.1477	0.4357	1.0000	0.6070	0.4357
W2V_SKIP_400	0.1401	0.2031	0.1658	0.0904	0.3929	1.0000	0.5641	0.3929	0.2229	0.2885	0.2515	0.1438	0.4214	1.0000	0.5930	0.4214
W2V_SKIP_500	0.1355	0.1978	0.1609	0.0875	0.3571	1.0000	0.5263	0.3571	0.2259	0.2913	0.2545	0.1458	0.4143	1.0000	0.5859	0.4143
W2V_SKIP_600	0.1416	0.2048	0.1674	0.0914	0.3643	1.0000	0.5340	0.3643	0.2214	0.2871	0.2500	0.1429	0.4143	1.0000	0.5859	0.4143
W2V_SKIP_700	0.1355	0.1978	0.1609	0.0875	0.3500	1.0000	0.5185	0.3500	0.2108	0.2772	0.2395	0.1361	0.4071	1.0000	0.5787	0.4071
W2V_SKIP_800	0.1325	0.1943	0.1576	0.0855	0.3571	1.0000	0.5263	0.3571	0.2018	0.2685	0.2304	0.1302	0.4143	1.0000	0.5859	0.4143
W2V_SKIP_900	0.1310	0.1925	0.1559	0.0845	0.3571	1.0000	0.5263	0.3571	0.2048	0.2715	0.2335	0.1322	0.4000	1.0000	0.5714	0.4000

In "Japanese words and English acronyms," fastText with skipgram performed well on all indicators. In particular, the 400-dimensional model showed the best values for precision, F1-score, and accuracy at 0.4643, 0.6341, and 0.4643, respectively, For fastText with CBOW at 400 dimensions, these indices increased by about 0.25, while for the other fastText with skipgram, they increased by about 0.11 to 0.13.

### Analysis for eight fields in radiological technology

In the evaluative indices, the fields of “Image Engineering”, “Physical Phenomena”, “Equipment”, “Radiation Therapy”, “Medicine”, and “Imaging Diagnosis” manifested optimal values when processed with fastText with CBOW. In these fields, the optimal vector dimensionality was consistently below 500, gravitating towards approximately 300 dimensions. Specifically, within the realm of “Imaging Diagnosis”, it emerged as the most superior among all categories. The recorded values for precision, recall, F1-score, and accuracy were 0.7137, 0.9586, 0.8182, and 0.6923, respectively. The "Radiation Control" showed optimal performance when processed in fastText using the skip-gram approach at 600 dimensions. The "Informatics" showed the best values in all evaluation metrics when subjected to Word2vec with CBOW at 800 dimensions. In the investigation of Japanese notation patterns, it was observed that plural expressions exceeded 30% across all domains apart from Radiation Therapy. In the fields of Equipment, Informatics, and Radiation Therapy, the frequency of “conversion to transliteration” was notably higher, accounting for approximately 20% or more in comparison to other categories. “Japanese shortened forms” were prevalent, constituting over 20% within the domains of Medicine and Imaging Diagnosis. Moreover, “Different Japanese spellings with the same meaning” appeared most frequently in the areas of Physical Phenomena, Radiation Control, and Physical Phenomena again, exceeding 40% (Table 9).

**Table 9:** The percentage of each Japanese expression pattern in the eight fields.

Field	Transliteration variants	Different Japanese spellings with the same meaning	Japanese shortened forms	Conversion to transliteration	Plural expressions
Image engineering	1.4	22.8	6.9	8.3	60.7
Physical Phenomena	0	44.9	7.1	11.7	36.2
Radiation control	0	43.1	11.1	6.9	38.9
Equipment	2.4	17.9	4.2	26.8	48.8
Informatics	3.4	29.2	2.2	24.7	40.4
Radiation therapy	0	43.1	21.6	19.6	15.7
Medicine	8.1	32.4	17.6	8.1	33.8
Imaging diagnosis	1.7	17.9	21.4	9.8	49.1

## Discussion

### Comparison between Word2vec and fastText

Across all indices, fastText consistently outperformed Word2vec. Notably, fastText employing the CBOW architecture peaked in performance at 300 dimensions. Furthermore, scores remained relatively stable across various vector dimensions within fastText with CBOW. The disparity between the maximum and minimum values was a mere 0.03, translating to a difference of about 30 words attributable to variations in the number of vector dimensions. In the context of Word2vec, models crafted using the skip-gram approach outpaced those based on CBOW, with the exception of the 100-dimensional representation. Given the outcomes, it is evident that the most effective architecture for synonym extraction within the domain of radiation technology is the fastText model utilizing the CBOW approach, with vector dimensions ranging between 300 and 400. However, considering the tendency for synonyms to appear in lower ranks, effective automation would require extracting terms from multiple ranks and implementing a robust filtering mechanism to refine the synonym selection.

### Analysis for the synonym expression patterns

**Figure 5:**
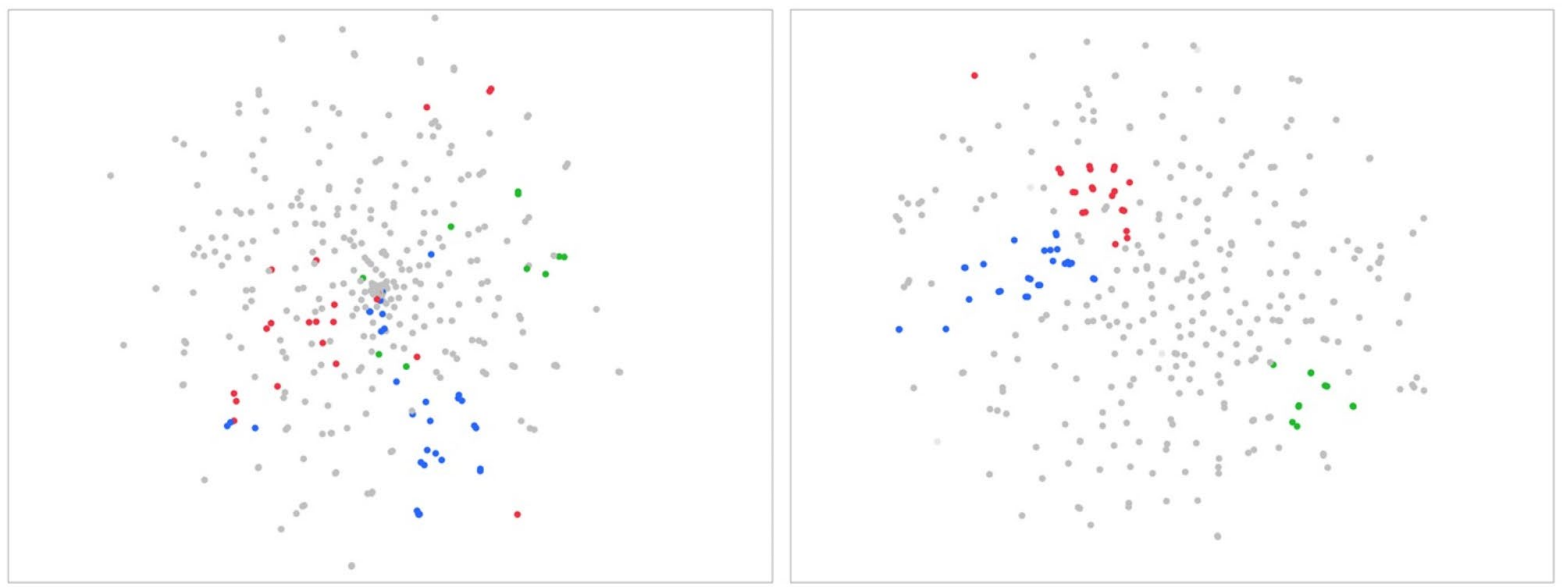
t-SNE map of terms in synonym sets. The left panel is for Word2vec with CBOW and 800 vector dimensions, and the right panel is for fastText with CBOW and 400 dimensions. Words are sets in “shortened Japanese.” Green words include “irradiation(照射)” or “irradiation method(照射法).” Blue words show “contrast　(造影)” or “contrast method　(造影法).” Red words show “-graphy (撮影)” or “-graphy method (撮影法).”

In the evaluation of seven expression patterns, fastText outperformed word2vec in four categories: “transliteration variants,” “different Japanese forms with the same pronunciation and meaning,” “Japanese shortened forms” and “plural expressions.” A common feature of these four categories of synonym sets was the few differences of the number of characters in words in sets. Expressed differently, synonyms in these categories had a common n-gram. Figure 5 shows the distribution of word vectors by t-distributed stochastic neighbor embedding (t-SNE) which is an unsupervised dimension reduction technique^30^. Here we will focus on the sets of “Japanese shortened forms”. In fastText, terms with the same n-gram tend to be located closer. Clusters of words including those with the same n-gram tends to spread widely and overlap in Word2vec. This result also suggests that fastText is advantageous in detecting synonyms with the same n-gram in high rankings.

The optimal architecture (CBOW and skip-gram) and the number of vectors in these four categories differed depending on the categories of the synonym sets. For the architecture, skip-gram was adequate only for "different Japanese forms with the same pronunciation and meaning". In fastText and CBOW the tendency is to use words containing a common n-gram as synonyms, and skip-gram tended to extract synonyms pairs that did not have a common n-gram. The more sets that include a common n-gram, the more CBOW would be advantageous, and if not, it may be equivalent to skip-gram or skip-gram may be advantageous. For vector dimensions, the ratio was improved or did not change significantly when the dimension number was larger. As mentioned in previous studies^16,28^, it has been reported that the accuracy improves as the vector dimensions increases. However, the optimum vector size may change depending on the characteristics of the synonym sets. This matter is also a subject for future investigations.

In “conversion to transliteration”, “Japanese words and English acronyms” and “Japanese and English words”, four indices in word2vec were equivalent or better when compared to the models by fastText, depending on the number of vector dimensions. However, the accuracy was the highest, with 50% in “Japanese words and English acronyms” and less than 30% in the others, which was inferior to that in the above four categories. Synonym sets in these categories had few or no common character strings, and it is difficult for fastText to perform well, making it necessary to consider ways to improve the accuracy of Word2vec.

It has been observed that when generating outputs involving English words and abbreviations, Japanese words tend not to rank at the top. This phenomenon is likely attributable to the models being predominantly trained on a Japanese corpus, thereby limiting their exposure to sufficient English expressions. Notably, the accuracy for both English words and abbreviations improved significantly when the models were constrained to output only alphabet characters. This finding suggests that specifying character sets can be a beneficial strategy when aiming to generate outputs in a language different from that of the training corpus.

### Synonym expression patterns

In the domains of “Image Engineering,” “Physical Phenomena,” “Equipment,” “Radiation Therapy,” “Medicine,” and “Imaging Diagnosis,” optimal outcomes were observed when employing FastText with the CBOW model. It was noted that in these fields, synonyms commonly included words with shared n-grams. Conversely, in the areas of Radiation Control and Imaging Diagnosis, the fastText model utilizing Skip-grams was favored. This preference could be attributed to the model”s enhanced capability in detecting words that posed challenges for the fastText model with CBOW, thereby potentially contributing to the improved performance in evaluation indices. In the field of “Informatics,” a considerable number of synonyms were categorized under “different Japanese writings of the same meaning” and “Japanese word and Japanese transliteration.” These categories were distinguished by a markedly lower frequency of shared n-grams. In scenarios characterized by a reduced prevalence of common n-grams among synonyms, the Word2vec model might exhibit a comparative advantage over fastText.

### Comparison with previous studies

When contrasting the skip-gram and CBOW architectures within Word2vec, several studies have indicated a superior performance by CBOW in tasks like similar word detection and text classification^31,32^. In the context of fastText, existing research has posited that skip-gram surpasses CBOW, especially in sentiment analysis-based classifications^33,34^. The outcomes from our research align with these findings for Word2vec. In our study, however, the difference in the cumulative ratio between the most accurate model (CBOW) and skip-gram was marginal, at approximately 1.9%, as detailed in Table 4. This marginal difference could hint at an inherent advantage for skip-gram depending on the nature of the task at hand.

Regarding the dimensionality of word embeddings, various studies have explored the relationship between accuracy and vector dimensions. For instance, Milolov et al. described that the accuracy increased as vector size increased^26^. Concurrently, Pennington et al. highlighted a peak in accuracy around the 300-dimensional mark^35^. Our results resonate with these observations, underscoring the general trend seen in word embedding research. However, it’s worth noting that these prior studies didn’t exclusively target medical terminology and differed in their specific tasks compared to our research.

## Limitations

The synonym set employed in this study was curated by two experts drawing from a glossary provided by academic societies. However, this approach is not impervious to potential omissions. Additionally, as part of the preprocessing, spaces were introduced between words in Japanese text using the morphological analysis tool. A significant challenge in morphological analysis is the handling of out-of-vocabulary (OOV) words. When these OOV words, not covered in the dictionary, are subjected to analysis, there is a risk of incorrect segmentation. This becomes especially challenging when the OOV (Out of Vocabulary) word is a technical term, as it may hinder the successful identification of synonymous candidates. Due to the collected text in the learning corpus primarily focusing on “image diagnosis”, there is a possibility that adequate learning has not been achieved for areas with low relevance, such as radiation measurement.

## Conclusions

The application of Word2vec and fastText models for automatic synonym detection in the field of radiological technology indicated that the fastText with CBOW at 300 dimensions was the most precise. In the detailed analysis of synonym notation patterns, it was found that fastText with CBOW excelled in cases where synonyms shared common n-grams. Conversely, fastText with skip-gram and Word2vec with CBOW models were more effective in instances where synonyms did not share common n-grams. In the eight fields pertinent to radiological technology, the fastText with CBOW model proved particularly beneficial due to the frequent occurrence of common n-grams. However, in the field of informatics, where English terms, acronyms, and transliterations are commonly employed, the Word2vec model with CBOW architecture showed greater utility.

## Data Availability

The datasets used and/or analysed during the current study available from the corresponding author on reasonable request.
